# Through the HoloLens™ looking glass: augmented reality for extremity reconstruction surgery using 3D vascular models with perforating vessels

**DOI:** 10.1186/s41747-017-0033-2

**Published:** 2018-01-31

**Authors:** Philip Pratt, Matthew Ives, Graham Lawton, Jonathan Simmons, Nasko Radev, Liana Spyropoulou, Dimitri Amiras

**Affiliations:** 1 Department of Surgery and Cancer, Imperial College London, London, UK; 2 Department of Plastic and Reconstructive Surgery, Imperial College Healthcare NHS Trust, London, UK; 3 Hamlyn Centre for Medical Robotics, Imperial College London, London, UK; 4 Imperial College School of Medicine, Imperial College London, London, UK; 5 Department of Radiology, Imperial College Healthcare NHS Trust, London, UK

**Keywords:** Augmented reality, HoloLens, Three-dimensional (3D) reconstruction, Vascular pedicle flap, Computed tomography

## Abstract

Precision and planning are key to reconstructive surgery. Augmented reality (AR) can bring the information within preoperative computed tomography angiography (CTA) imaging to life, allowing the surgeon to ‘see through’ the patient’s skin and appreciate the underlying anatomy without making a single incision. This work has demonstrated that AR can assist the accurate identification, dissection and execution of vascular pedunculated flaps during reconstructive surgery. Separate volumes of osseous, vascular, skin, soft tissue structures and relevant vascular perforators were delineated from preoperative CTA scans to generate three-dimensional images using two complementary segmentation software packages. These were converted to polygonal models and rendered by means of a custom application within the HoloLens™ stereo head-mounted display. Intraoperatively, the models were registered manually to their respective subjects by the operating surgeon using a combination of tracked hand gestures and voice commands; AR was used to aid navigation and accurate dissection. Identification of the subsurface location of vascular perforators through AR overlay was compared to the positions obtained by audible Doppler ultrasound. Through a preliminary HoloLens-assisted case series, the operating surgeon was able to demonstrate precise and efficient localisation of perforating vessels.

## Key points

- Augmented reality can demonstrate subsurface vascular anatomy before incisions are made.
- Manual alignment is sufficiently fast and accurate to guide the operative incision.
- Automated registration would further open access of the HoloLens in clinical use.

## Background

This work presents a series of pioneering attempts to utilise augmented reality (AR) technology in reconstructive surgery with the HoloLens™, an AR device developed and marketed by the Microsoft Corporation (Redmond, WA, USA). It aims to test the ability of this wearable system to aid the identification of surgical landmarks when performing vascular pedunculated flaps of the lower extremities. Three-dimensional (3D) virtual reality has already been shown to be useful in the identification of suitable perforators in deep inferior epigastric artery perforator flaps and improves perioperative outcomes [1, 2]. The intraoperative application of AR offers exciting possibilities, such as the simplification and accurate performance of procedures that could result in the reduction of anaesthetic time and occurrence of adverse outcomes. Furthermore, it can facilitate preoperative planning and surgical training, as well as provide 3D telemedicine support.

One of the key advantages of wearable systems such as the HoloLens is that it can be used without compromising environment sterility. It is operated with hand gestures and voice commands instead of touch. As a self-contained computer, a very broad range of information can be accessed in real time while remaining sterile in the operating theatre. Novel techniques projecting reconstructed computed tomography (CT) data have shown benefit in the localisation of lymph nodes and planning of flaps [3, 4]. What is more, it is believed that reconstructive surgery provides an excellent opportunity for AR in the visualisation of tissue following injury, when anatomical landmarks might have been distorted. In addition, accurate detection of complex vasculature is of paramount importance in the performance of vascular flaps and AR can enable detailed yet unambiguous visualisation to help avoid potential errors. Furthermore, the lower extremities permit the use of relatively rigid geometrical structures, such as bony prominences and the skin silhouette, which help to overcome the main registration limitation of tissue deformation.

The HoloLens was chosen as it is currently considered to be one of the most suitable AR devices for surgical practice [5, 6]. CT angiography (CTA) has been shown to reduce operative time by allowing visualisation of potential flap perforators and for comparison to be made between perforators in terms of their suitability [7, 8]. CTA may also reduce the incidence of partial and complete flap loss [9] and its superiority over Doppler ultrasound is well established [10]. Furthermore, CTA is a useful technique to examine the donor vascular anatomy or the vascular supply to an injured extremity [11].

Some of the drawbacks of using CTA stem from transferring the elaborate imaging information into a clinically useable form and thence the availability of this information during an operation. Often this requires the surgeon to pre-examine the scans in detail using measurements of a perforator’s location from several anatomical landmarks [12]. The surgeon must then triangulate the position of that perforator on the patient at the outset of the procedure, without any depth analysis. This is not only time-consuming and complex, especially when dealing with multiple perforators, but also error-prone. Other techniques such as stereotactic image-guided navigation [13] and combining CT, Doppler ultrasound and radio-opaque markers have been described [14].

AR offers a novel solution to the problem of accurate and rapid perforator localisation by allowing image overlay on the patient during the operation. In a patient-specific manner, relevant information can be built into the rendered ‘hologram’ with varying degrees of complexity. Thus, multiple perforators, their source, course and relation to the underlying skeleton and nearby wounds can be immediately identified. This enables the surgeon to tailor their approach according to the specific anatomical variations of the patient. With that goal in mind, the following sections outline use of the HoloLens system during the development of perforating blood vessel maps in plastic surgery.

## Methods

**Fig. 1:**
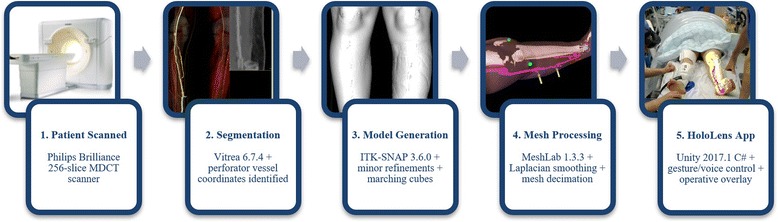
Workflow diagram showing the processes involved in AR content production

The overall workflow is summarised in Fig. 1. Written informed consent was taken from patients recruited to this ethically approved preliminary study. The HoloLens software was used only within the institution where it was developed. Preoperative contrast-enhanced CTA scans were performed using a 256-slice Philips Brilliance CT scanner (Koninklijke Philips N.V., Amsterdam, The Netherlands). The scans were undertaken with patients in the prone position to limit tissue deformation of the soft tissues of the calf and to reduce compression of the perforating veins. In certain cases, this was not possible due to other injuries limiting prone positioning of the patients. As an alternative, the respective legs were elevated to prevent compression of the calf skin and muscle. This was done to mitigate the effect of anatomical deformation between acquisition time and intervention and thereby minimise registration error. In order to distend the lower leg perforators, a tourniquet was applied above the knee level. To obtain both arterial and venous phases within a single contrast scan, a phased administration of contrast agent was performed. Specifically, a split bolus comprising two 70-mL volumes of Omnipaque (General Electric Healthcare, Chicago, IL, USA) was administered intravenously, followed by a saline bolus chaser. Typically, the images were acquired with an axial in-plane resolution of 0.7 mm and slice thickness of 0.9 mm.

The CTA volumes were segmented using the Vitrea 6.7.4 software (Vital Images, Inc., Minnetonka, MN, USA) into skin, bone, muscle, and vascular models by the consultant radiologist. The vascular models were produced with segmentations of both venous and arterial vessels of the lower leg. Where appropriate, the trunks of the medial sural artery perforators (MSAPs) were segmented together with the associated site of perforation through the muscle fascia. The lengths of the trunk and its branches were calculated using the Vitrea built-in vascular tools and the approximate site of each perforator was measured relative to the medial femoral epicondyle. Skin segmentations were performed using the Vitrea *autoskin* function, whereby a combination of voxel thresholding and morphological operations rapidly identifies the outer tissue layer. This facilitates accurate hands-free registration of models to their respective patients. In addition, the bone segmentations allowed for accurate correlation with anatomical landmarks such as the tibial tuberosity. Before surgery, each case was discussed with the surgical team to explain the models and anatomy and to confirm the choice of perforator. It was not uncommon to mark a selection of perforators so that a decision could be made at the time of surgery.

The segmented volumes were then loaded as Digital Imaging and Communications in Medicine (DICOM) files into ITK-SNAP 3.6.0 [15], where minor refinements were made through meticulous use of its region growing and manual painting functionality, and where mesh representations were generated by the marching cubes algorithm [16]. These were further manipulated with MeshLab 1.3.3 [17] for smoothing and mesh complexity. Respectively, the manipulations performed were Humphrey’s Classes Laplacian smoothing [18], followed by edge collapse decimation using a quadric error metric [19]. These resulted in optimised anatomical representations, increasing the performance of the HoloLens application while minimising loss of model precision. Tests were performed to check whether the loss of precision was within the clinically acceptable range (5 mm) for this specific application. For example, the axial length of the skin model for *Case #1* was reduced by 0.92 mm over a total of 591.6 mm, equal to a change of 0.16%.

Written within the Unity framework, version 2017.1 (Unity Technologies, San Francisco, CA, USA), a custom-developed HoloLens C# Universal Windows Platform application was utilised at the time of patient marking. Once launched, the application generated ‘holographic’ overlays, correctly rendered for both left and right eyes, at a default distance and rotation with respect to the wearer’s coordinate frame. Subsequently, using a combination of spatial translation and rotation hand gestures, the operating surgeon manipulated the virtual anatomy from a static posture until a satisfactory degree of anatomical landmark and skin outline alignment against the anaesthetised patient were achieved.

Through either voice commands or a toolbar button, the user interface permits switching between translation and rotation modes. In both cases the HoloLens ‘air-tap and hold’ gesture was used as a source of 3D motion input. Having been employed successfully in other image guidance applications [20], the ‘rolling ball’ control mechanism [21] was adopted as it provides a very intuitive way of manipulating orientation that is independent of observer viewpoint. The spatial scale information embedded in each CTA scan was retained throughout the segmentation and object building process, so that no scale adjustments were required following HoloLens model import. The required procedural changes were minimal. Having annotated the skin with a sterile marker pen under HoloLens guidance, the target positions were compared to the sites of the perforator vessels as subsequently identified by audible Doppler ultrasound and surgical appearances.

## Case series illustration

**Fig. 2:**
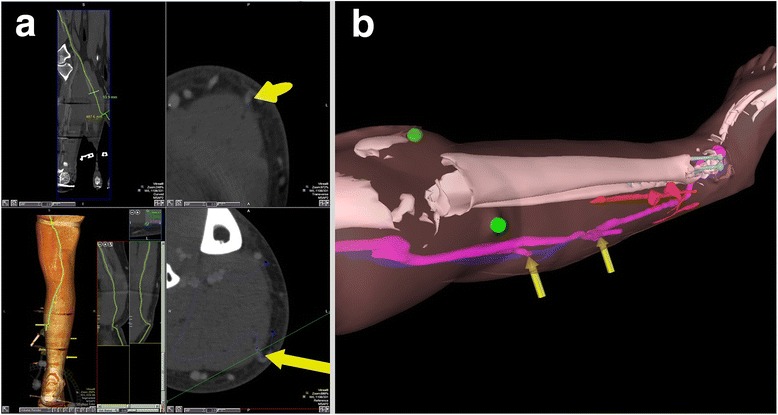
**a** Case 5 CTA imaging showing the location of perforating arteries with *yellow arrows*. **b** Case 2 example HoloLens rendering of segmented polygonal models

**Fig. 3:**
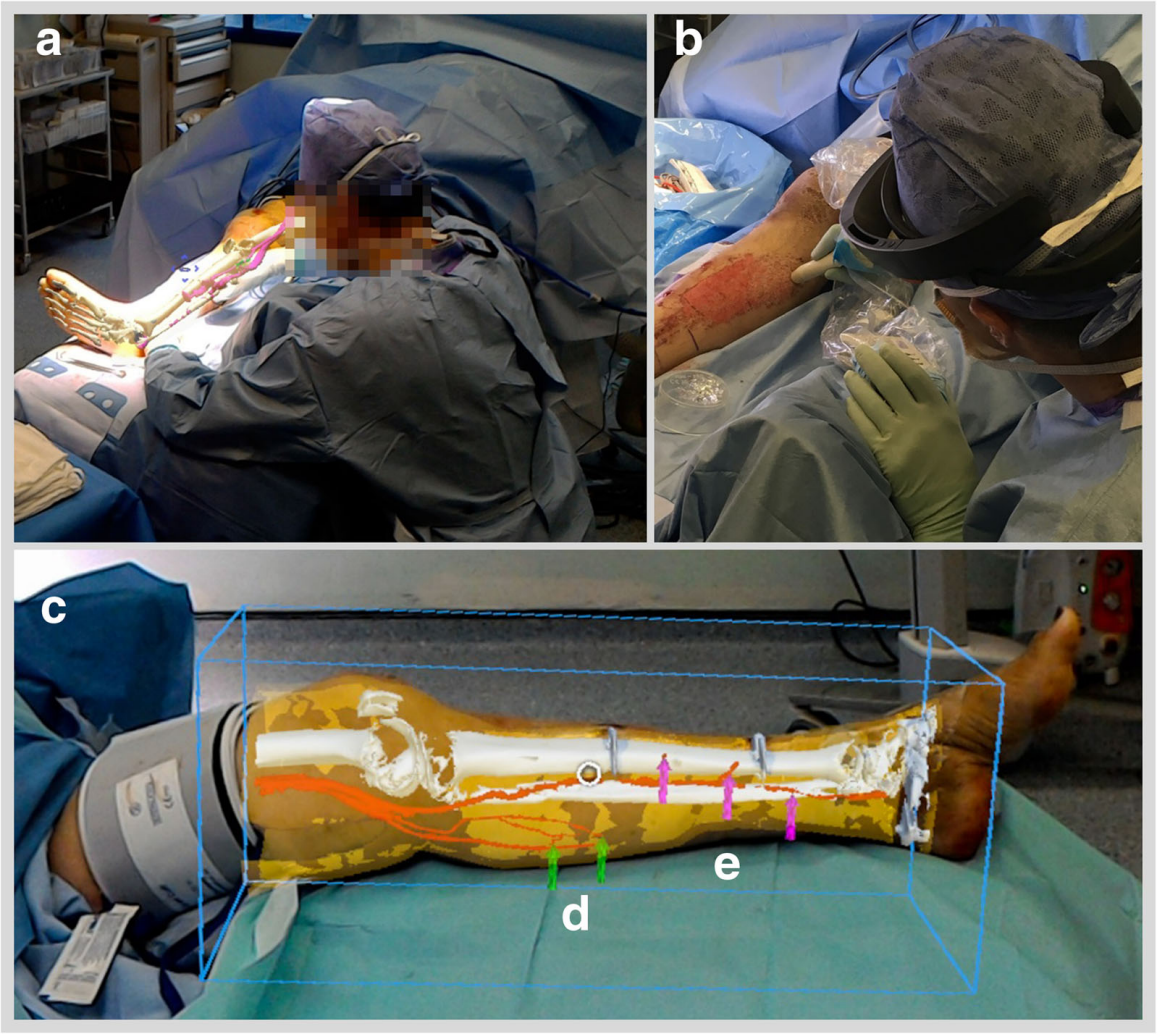
**a** Case 3 AR overlay of models as viewed from remote HoloLens; (**b**) confirmation of perforator location with audible Doppler ultrasonography. **c** Case 6 overlay with bounding box; *arrows* highlighting position of (**d**) medial sural and (**e**) posterior tibial perforators

**Fig. 4:**
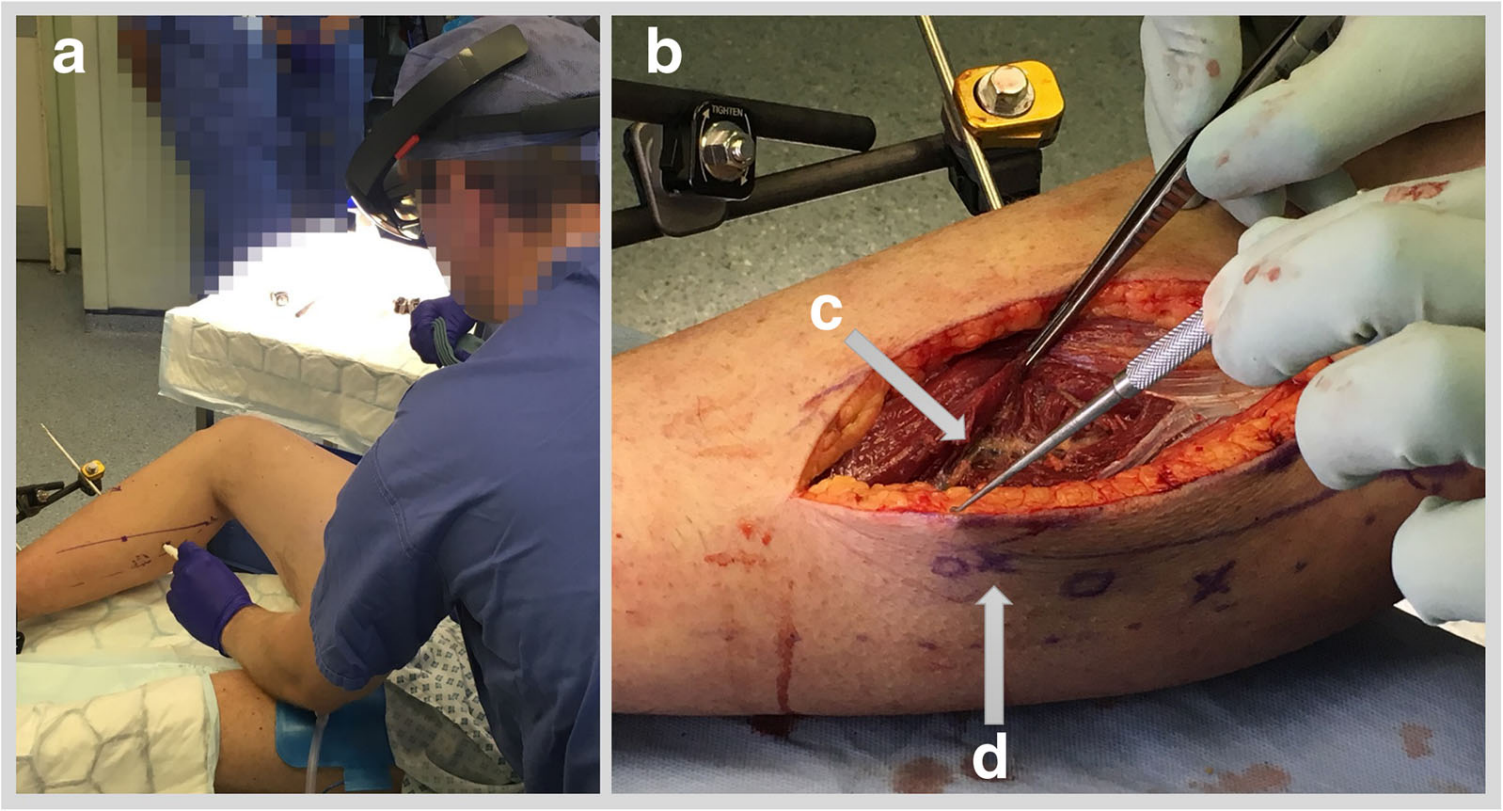
**a** Case 5 marking of skin under HoloLens guidance. **b** Case 5 raising of free flap commences; (**c**) dissection of perforating vessels and underlying vessels; (**d**) corresponding skin marking confirming registration accuracy

Table 1 summarises details of the case series. Five flap surgeries were performed using the medial sural artery perforators or other suitable donor sites. In one instance (Case 2), at the time of operation, the defect was covered with a split skin graft only. An example of the original CTA imaging, segmentation, and corresponding polygonal models is shown in Fig. 2. Captured from a HoloLens position remote from the patient, examples of AR overlay are shown in Fig. 3, adjacent to an image illustrating confirmation of target position with audible Doppler ultrasound. Figure 4 shows both the manner in which target positions within AR overlays are transferred to the skin permanently with a marker pen and the subsequent raising of the pedunculated flap. The approximate times taken to perform the steps comprising the workflow are summarised as follows: patient scan (5 min); segmentation (10–20 min); model preparation (20–30 min); HoloLens upload and configuration (<1 min); and intraoperative manual registration (1–2 min).

**Table 1:** Case series comprising six patients undergoing reconstructive surgery

Case	Gender	Age (years)	Perforator(s)	Flap type	Injury site	Target vessels
1	M	53	Medial sural artery	Free perforator with small muscle cuff	Lateral malleolus	Anterior tibial artery/vein
2	F	27	Medial sural artery	Split skin graft only	Lateral malleolus	Not applicable
3	M	57	Posterior tibial artery	Fasciocutaneous propeller	Medial malleolus	Not applicable
4	M	71	Posterior tibial artery	Free perforator	Distal lower leg	Posterior tibial artery/vein
5	M	41	Medial sural artery	Free perforator	Lateral malleolus	Anterior tibial artery/vein
6	F	85	Posterior tibial artery	Fasciocutaneous propeller	Medial malleolus	Not applicable

### Case 1

This 53-year-old man sustained an open subtalar joint dislocation, with loss of soft tissue over the lateral aspect of the foot and ankle. The MSAP flap was chosen due to the thin pliable tissue being a good match with the native tissue and its lack of bulk. Intraoperatively, a 20 × 15 cm flap was raised based on a perforator at 13 cm from the joint line. The position of the MSAP was identified using the HoloLens overlay and traditional Doppler technique. The surgical position of the MSAP was visually indistinguishable from that identified by the HoloLens. Subsequently, the distal 2 cm of the pedicle was raised with a narrow cuff of muscle.

### Case 3

This 57-year-old man sustained an open fracture of the medial malleolus of his right ankle following a road traffic accident. He was worked up for a free tissue transfer to cover this as there were no obviously good local tissue options. A very large perforator was noted coming off the posterior tibial artery proximal to the defect. The decision was made therefore to proceed with a propeller flap. The perforator was completely dissected out down to the deep fascia and the position of the perforator corresponded to the position identified by the HoloLens with no visible discrepancy. The deep fascia and the proximal tissue were then rotated 180° into the soft tissue defect.

### Case 4

Following a previously treated open tibial fracture one year before the current presentation, this patient suffered a minor cut which later developed into infection, requiring formal soft tissue reconstruction. The patient was worked up for a MSAP flap from the contralateral side as, due to the extensive previous trauma, it was felt that a traditional distally based local flap was not possible. However, CTA revealed two good-sized ipsilateral tibialis posterior perforators entering the soft tissue proximal to the defect. On this occasion, there was interval removal of external fixation that resulted in soft tissue deformation and a < 1-cm discrepancy in the operative AR position of the perforator and the surgical findings. A fasciocutaneous flap was then fashioned that was transposed distally over the defect to gain good soft tissue cover.

### Case 5

This 41-year-old man was involved in a road traffic accident and sustained a multiplanar degloving to the lateral aspect of his right foot and ankle, with an exposed open displaced dislocation of the talus. There was a defect in the soft tissue underlying the bone which had been stripped of much of its periosteum and therefore it was concluded that he would require flap coverage. In accordance with the CTA findings and HoloLens overlay, a MSAP was raised from the ipsilateral calf, 8 × 16 cm in size. The flap was anastomosed to the anterior tibial artery with an end-to-side join. A 2-mm coupler was used to join a vena comitans to the anterior tibial vein in an end-to-end fashion. The short saphenous vein that was harvested within the flap was anastomosed to a superficial vein within the foot.

### Case 6

An 85-year-old woman sustained an open medial malleolus fracture with an associated closed fibular fracture. Following an initial debridement procedure, the patient returned to theatre where a lateral plate was used to fix her fibula. The medial wound was too large to close directly and thus a fasciocutaneous flap was raised from her medial lower leg adjacent to the defect. While three posterior tibial perforators were seen on the CT angiogram and visualised with the HoloLens, the flap was established to include the two most inferior vessels. Doppler ultrasound confirmed accurate localisation with no visible discrepancy. While optimising blood supply, the two-vessel solution did slightly restrict movement, although sufficient transposition of tissue was ultimately possible.

## Discussion

It was possible to construct valuable AR models from CTA scans of the lower leg perforators and to use these models in a series of reconstructive surgeries over six cases. The HoloLens proved to be a powerful tool that has the potential to reduce anaesthetic time and morbidity associated with surgery as well as to improve training and provide remote support for the operating surgeon. Detailed feedback from the surgical team verified that this new approach is more reliable and therefore considerably less time-consuming than audible Doppler ultrasound, the prevailing standard method of navigation. The specific challenges addressed in this preliminary report include the provision of a practical user interface for spatial model manipulation, streamlining of the data preparation pipeline for high-resolution volumes and the export of reference coordinates for arrow localisation. One limitation is that presently a technical assistant is required initially to help with preoperative data preparation and later in the operating theatre to assist with application launch and approximate spatial model positioning before involvement of the operating surgeon.

Further work is certainly warranted to realise automatic segmentation, volumetric rendering and instantaneous model alignment, to correct for tissue deformation, to measure the impact on operative time and surgical outcomes, and to quantify further the targeting accuracy with respect to traditional methods. The experience gained hitherto suggests that the techniques developed through this work are appropriate for reconstructive surgery applied to other areas of the body.

## Abbreviations

3D: Three-dimensional
AR: Augmented reality
CT: Computed tomography
CTA: Computed tomography angiography
DICOM: Digital Imaging and Communications in Medicine
MSAP: Medial sural artery perforator
